# Evaluating health policies with subnational disparities: a text-mining analysis of the Urban Employee Basic Medical Insurance Scheme in China

**DOI:** 10.1093/heapol/czac086

**Published:** 2022-10-11

**Authors:** Kai Liu, Wenting Liu, Alex Jingwei He

**Affiliations:** School of Labor and Human Resources, Renmin University of China, No. 59, Zhongguancun Street, Beijing, China; Department of Social Work, The Chinese University of Hong Kong, Shatin, New Territories, Hong Kong SAR, China; Department of Asian and Policy Studies, The Education University of Hong Kong, 10 Lo Ping Road, Tai Po, New Territories, Hong Kong SAR, China

**Keywords:** Health policy evaluation, subnational disparity, text mining, Urban Employee Basic Medical Insurance, China

## Abstract

Subnational disparities in most health systems often defy ‘one-size-fits-all’ approach in policy implementation. When local authorities implement a national policy in a decentralized context, they behave as a strategic policy actor in specifying the central mandates, selecting appropriate tools and setting key implementation parameters. Local policy discretion leads to diverse policy mixes across regions, thus complicating evidence-based evaluations of policy impacts. When measuring complex policy reforms, mainstream policy evaluation methodologies have tended to adopt simplified policy proxies that often disguise distinct policy choices across localities, leaving the heterogeneous effects of the same generic policy largely unknown. Using the emerging ‘text-as-data’ methodology and drawing from subnational policy documents, this study developed a novel approach to policy measurement through analysing policy big data. We applied this approach to examine the impacts of China’s Urban Employee Basic Medical Insurance (UEBMI) on individuals’ out-of-pocket (OOP) spending. We found substantial disparities in policy choices across prefectures when categorizing the UEBMI policy framework into benefit-expansion and cost-containment reforms. Overall, the UEBMI policies lowered enrollees’ OOP spending in prefectures that embraced both benefit-expansion and cost-containment reforms. In contrast, the policies produced ill effects on OOP spending of UEBMI enrollees and uninsured workers in prefectures that carried out only benefit-expansion or cost-containment reforms. The micro-level impacts of UEBMI enrolment on OOP spending were conditional on whether prefectural benefit-expansion and cost-containment reforms were undertaken in concert. Only in prefectures that promulgated both types of reforms did UEBMI enrolment reduce OOP spending. These findings contribute to a comprehensive text-mining measurement approach to locally diverse policy efforts and an integration of macro-level policy analysis and micro-level individual analysis. Contextualizing policy measurements would improve the methodological rigour of health policy evaluations. This paper concludes with implications for health policymakers in China and beyond.

**Key messages**

- Studies evaluating the impacts of health policies typically adopt simplified policy proxies, overlooking distinct policy choices across localities in decentralized settings.
- We develop a novel text-mining approach to measuring locally diverse policy efforts and apply it to health policy evaluation.
- A case study on the Urban Employee Basic Medical Insurance in China suggests that the program reduced individual out-of-pocket health spending more efficiently in prefectures that synchronize multiple reforms than those implementing poorly coordinated reforms.
- Contextualizing policy measurement could improve the methodological rigour of health policy evaluation when subnational policy disparities are evident.

## Introduction

Healthcare reforms often defy the one-size-fits-all paradigm, especially in large countries with wide subnational disparities. Decentralization accounts well for such disparities and offers necessary discretion and flexibility in implementation and even in policy designs in the health sector (Bossert and Beauvais, 2002). When implementing national policies in a decentralized system, local governments are often granted a certain degree of autonomy in adjusting the policy package, selecting the timing of the launch and deploying implementation instruments (Bossert and Mitchell, 2011; Brixi *et al.*, 2013). When a national health policy reform encompasses multiple interventions, local authorities commonly have the discretion to selectively adopt the policy package and decide which interventions to prioritize in their implementation agenda, thereby creating diverse policy mixes across localities (Liu *et al.*, 2022). In this regard, subnational governments do not operate merely as passive policy implementers but may instead behave as strategic policy actors when national policy mandates come from above. In reality, they are often found to balance top-down policy instructions and local interests in a pragmatic and skilful way. The literature, however, falls short in explaining the impact of locally diverse policies on individual well-being.

Developing countries, in their pursuit of universal health coverage, oftentimes undertake multiple health policies simultaneously (Bali and Ramesh, 2019; Chu *et al.*, 2019), thus complicating the isolation of net intervention effects. Health financing reforms, e.g., seek to provide financial protection through two policy mechanisms: universal risk pooling and strategic purchasing (WHO, 2010). A health insurance program may reduce individual out-of-pocket (OOP) payments by increasing the proportion of insurance payout and expanding service coverage (the risk-pooling mechanism). In the meantime, a health insurance program may improve the efficiency of insurance funds by paying providers in a strategic manner that maximizes the value of health insurance and promotes quality assurance of health services (the strategic purchasing mechanism). Unfortunately, many existing studies tend to evaluate such big health policy programs by relying predominantly on simplified policy measures that mask the innate complexities of large-scale interventions, and thus they have offered little convincing evidence regarding the specific mechanisms through which big policies affect individual-level outcomes (Liu *et al.*, 2021).

Using the emerging ‘text-as-data’ methodology (Grimmer and Stewart, 2013; Altaweel *et al.*, 2019), this study developed a novel approach to policy measurement that is applicable to health policy evaluation. Taking the case of the Urban Employee Basic Medical Insurance (UEBMI), the flagship social health insurance (SHI) program in China, we established a diachronic health policy database that covered policies implemented by prefectural governments—the authorities that manage SHI in China—and we then used the technique of keyword analysis to capture multiple policy interventions. This analytic strategy allowed us to construct a set of new UEBMI policy measures that were capable of addressing both the detection of local policy disparities and the decomposition of specific policy mechanisms. By matching policy data with longitudinal survey data, we estimated the impacts of these policy measures on individual OOP health spending (OOP spending) at two levels. At the macro level, we examined the impact of diverse prefectural UEBMI reforms on individual OOP spending, while at the micro level, we investigated the heterogeneous impact of individuals’ UEBMI enrolment on OOP spending under different prefectural UEBMI policy conditions.

This study contributes to health policy research in two ways. First, the text-mining approach to policy measurement allows researchers to identify subnational disparities in the implementation of the same national health policy in a variety of regions. This methodological strategy contributes to a comprehensive measurement of locally diverse policy efforts of the same policy program in a country. Second, this study explores the ways of determining appropriate levels of analysis in health policy evaluation by distinguishing macro policies from micro individual analysis. A health policy is ontologically a macro-level entity and should be contextualized instead of reduced to individual participation when its impacts are evaluated. This study experiments the contextualization of policy measurement to improve the methodological rigour of health policy evaluation.

## Research background

The outcome of national health policies ultimately depends on their implementation at the local level, particularly in large countries. Depicted by the top-down conception of policy implementation, the old notion that local authorities are merely passive implementers of centrally mandated policies has been widely recognized to be problematic (Thomann *et al.*, 2018; Guo *et al.*, 2021). Subnational agents do own considerable discretion over many key parameters of implementation, including timing and the tools mix. Many factors have been found to influence local health policy choices, including intergovernmental relations, personal traits and career motivation of local leaders, local fiscal capacity, industrial structure and demographic characteristics (Ciani *et al.*, 2012; Boehmke *et al.*, 2017; Conti and Jones, 2017; Huang and Kim, 2020; Arlotti *et al.*, 2021).

A key stream of health policy research is concerned with the extent to which policy interventions lead to their intended outcomes (Adam *et al.*, 2012; Drummond *et al.*, 2015). Mainstream methodological protocols typically require an experimental or quasi-experimental design to evaluate the impact of policy interventions, and that design is expected to capture three essential components: policy treatments, outcome indicators and the identification of causality between them (Angrist and Pischke, 2009; Khandker *et al.*, 2010). The dominant methodological strategy in evaluation studies has focused on the second and third components, aiming to examine policy effects on a variety of potential outcomes, such as service utilization, health spending, patient satisfaction, clinical outcomes and health inequality (Aggarwal 2010; Finkelstein, 2007; Wagstaff and Lindelow, 2008; King *et al.*, 2009; Baicker *et al.*, 2013; Aryeetey *et al.*, 2016). That approach also addresses endogeneity, selection bias and confounding effects in identifying the causal effects, relying on econometric methods such as a difference-in-differences design, instrumental variables, propensity score matching and regression discontinuity design (Finkelstein, 2007; Wagstaff and Lindelow, 2008; Wagstaff *et al.*, 2009; Sommers *et al.*, 2012; Liu and Zhao, 2014; Aryeetey *et al.*, 2016; Bernal *et al.*, 2017).

However, when measuring policy interventions, these studies have tended to adopt a simplified measurement that may overlook local policy discretion and discrepancies and leave largely unknown the heterogeneous effects of the same policy’s implementation with different choices and specifics across regions. Specifically, most studies have used two simplified measures as health policy proxies. The first is a micro measure of individual-level policy coverage defined as whether or not an individual is covered by a policy program, regardless of its specific design or scheme content. Researchers have utilized such a binary typology of micro-level participation to identify the differences in outcome variables between policy beneficiaries and non-beneficiaries or to compare the changes of outcome variables before and after an individual participates in a policy program. Policy effects are commonly considered to be the average changes in outcomes that are driven by individual-level policy participation (Aggarwal 2010; Wagstaff and Lindelow, 2008; Bernal *et al.*, 2017; Huang and Gan, 2017). This approach, using micro policy measurement, replaces policy analysis with individual analysis, thus reflecting a reductionist orientation. This behaviourist way of thinking typically reduces collective phenomena to the aggregation of individual actions, motivations and preferences (Easton, 1985). Since the 1980s, many neo-institutionalists have criticized behavioural research for its reductionist methodology and its neglect of the autonomy of institutions, systems and policies (March and Olsen, 1984; Immergut, 1998).

The second type of oft-used policy proxy is a macro measure of regional-level policy adoption, which captures the region-specific timing of policy adoption. It is employed to assess the double differences in outcome indicators between implementing regions and non-implementing regions, as well as the outcome indicators between pre-implementation and post-implementation periods, thus resulting in an entangled estimate of policy effects (the so-called extensive margin). For instance, by identifying the temporal variations in adopting a specific health insurance policy among localities, researchers have examined the policy’s effects on a few outcome variables, such as health spending (Finkelstein, 2007; Wagstaff *et al.*, 2009), the probability of individual enrolment (Sommers *et al.*, 2012), service utilization (Liu and Zhao, 2014) and health outcomes (King *et al.*, 2009; Baicker *et al.*, 2013). Studies that use the timing of adoption to represent policies tend to treat policies after adoption as a monolith with no variation across localities, thus ignoring the fact that different regions may employ diverse strategies and tools to implement the same national policy. Such studies may also obscure various functions of a particular intervention, making it difficult to pinpoint the precise mechanisms and pathways through which a policy affects individual-level outcomes.

Given those limitations, it is necessary to develop a novel approach to measure diverse policy efforts across subnational regions. A text-mining analysis of policy documents can help researchers measure and evaluate local policy endeavours. Such practices have become feasible in recent years with the aid of big-data technologies and web crawler techniques. Some new research initiatives have leveraged web crawler or manual crawling to build policy or quasi-policy databases in the health policy arena (e.g. Jo and You, 2019; Debnath and Bardhan, 2020; Liu *et al.*, 2021).

Moreover, the rapid development of natural-language processing technologies has given rise to many new methods for policy analysis. Text-mining tools such as keyword analysis, latent Dirichlet allocation, structural topic modelling, sentiment analysis, naive Bayes algorithm and support vector machines have been widely used to uncover the strategic intent and latent patterns of a large unstructured corpus of texts (Grimmer and Stewart, 2013; Altaweel *et al.*, 2019; Jo and You, 2019; Liu *et al.*, 2021). In this study, we used web crawler techniques to collect health policy documents and an automated keyword analysis method to gauge local policy efforts.

## The UEBMI reforms in China

Before China’s market transition in 1980s, there were three health insurance programs in China: the Government Medical Insurance Scheme (GMI) for government and public sector employees, the Labor Insurance Scheme for employees of state-owned enterprises and the Cooperative Medical Scheme for rural residents (Wagstaff and Lindelow, 2008). However, the old financing regime was significantly weakened following structural changes in the economy. To protect people from financial risks associated with catastrophic health expenditures, the Chinese government launched three SHI programs incrementally: the UEBMI for urban employees primarily in the formal sectors; the New Cooperative Medical Scheme for rural residents and the Urban Resident Basic Medical Insurance for unemployed urban residents and those in the informal sectors.

Launched at the national level in 1998, the UEBMI provides mandatory coverage and comprehensive benefits to urban employees. The program is financed by contributions from both employers and employees, with former paying 6% of the payroll income and latter paying 2% of their monthly salaries. The entire premium paid by an employee, as well as a small fraction the employer’s payment, is deposited into an individual account, while the remainder is pooled to the social insurance fund. Local UEBMI offices define the scope of insurance benefits via a set of catalogues of medicines and health services. The local offices also establish complex rules for deductibles, reimbursement rates, copayment and ceilings. A notable reform was enacted in 2021 aiming to mitigate the financial stress on UEBMI by reducing the presence of individual accounts. The portion of employer’s contribution that previously went into individual accounts is now deposited into a new risk pool for outpatient services.

The UEBMI is associated with extensive local discretion in terms of both the timing of a local program’s launch and its design. First, the central government took an experimentalist approach to implementing the UEBMI by designating pilots in two prefectures in 1994. The pilot program was subsequently expanded to 57 prefectures in 1996, and the UEBMI was officially instituted by the central government in 1998. Given considerable discretion, several prefectures did not launch the program until 2003 for various reasons.

Second, once a prefecture launches its program, the government is responsible for formulating implementation strategies and operational protocols by considering the unique local circumstances, such as fiscal capacity and competitive pressure from peer governments (Liu *et al.*, 2022). In that decentralized context, the central authorities issued national guidelines to set the basic principles but did not dictate uniform rules for local implementation. Instead, local governments were entrusted to design specific implementation protocols. As a result, prefectural governments have had a considerable degree of discretion, leading to substantial variations in the UEBMI policies. Previous studies suggest that local health policy disparities in China may be driven by a set of socioeconomic and political factors, including local economic growth, fiscal capacity and autonomy, peer pressure, pressure from higher-level governments and interregional migration (Huang, 2015; Huang and Kim, 2020; Zhang, 2020; Liu *et al.*, 2022).

To analyse the diverse policy efforts across prefectures, we built an analytic framework and classified multiple UEBMI interventions into two categories: the expansion of benefit packages (‘benefit-expansion’ reforms) and cost-containment reforms, as shown in Table 1. The benefit-expansion category entails policy actions that seek to improve the depth of financial protection, and the cost-containment category refers to policy initiatives intended to curb cost inflation and thereby prevent financial risks associated with seeking care. This bidimensional framework corresponds with the World Health Organization’s proposal on moving towards universal health coverage: on the one hand, collecting and pooling adequate funds to provide sufficient financial protection; on the other hand, promoting efficient use of resources and eliminating waste (WHO, 2010).

**Table 1.** Analytic framework of the UEBMI policies

Category	Strategy of reform
Benefit-expansion reforms	Boosting coinsurance rates and maximum payments Broadening the scope of medicine catalogues Adopting supplementary programs
Cost-containment reforms	Promoting prospective payment methods Containing insurance fund expenses Negotiating medicine prices Improving the capacity of UEBMI agencies to manage contracted health facilities

Those two broad categories of policy initiatives have manifested in different stages of China’s healthcare reforms. Benefit-expansion reforms were prioritized at the early stage. Both the central and local governments invested heavily in increasing insurance benefits, adding new drugs and services into benefit catalogues and offering supplementary programs. The government anticipated that those benefit-expansion interventions would solve the longstanding issues of poor access to care and high OOP costs (Liu *et al.*, 2022). However, many studies suggested that benefit expansion alone was not enough to curb the rapid inflation of costs. If the multitude of perverse incentives on the supply side were to remain unchecked, a large portion of insurance benefits could easily be absorbed by profit-seeking providers, thus actually undermining the intended effect of financial protection on the demand side (Yip *et al.*, 2010; 2019; Ramesh *et al.*, 2014). Recognizing those deficiencies, the Chinese government responded by embarking on a series of new policy initiatives, particularly after the launch of the landmark national healthcare reform in 2009 (Yip *et al.*, 2012). One of those key initiatives was a gradual withdrawal from the cost-inflationary fee-for-service payment system, moving towards prospective payment mechanisms such as a global budget, capitation, per diem and diagnosis-related groups (He et al., 2022). Additional policy efforts have also been undertaken, such as revamping the drug pricing system and capacity building of SHI agencies (Liu *et al.*, 2017; Yip *et al.*, 2019).

## Methods

### Data sources

To evaluate heterogeneous UEBMI policies across localities, we established a Chinese health policy database by retrieving policy documents from official sources. First, we manually accessed 7041 official websites of central, provincial and prefectural governments, as well as major government departments in health affairs. Health-related policy documents were retrieved from these sources by using Python 3.7. A policy document was considered relevant if its title contained one of the Chinese characters referring to ‘medical’, ‘medicine’, ‘health’, ‘disease’, ‘hospital’, ‘clinic’, ‘outpatient’ or ‘hospitalization’. A total of 234 412 documents were obtained, 168 678 of which were issued by prefectural authorities. Second, by employing an automated text-mining tool called ‘Term Frequency-Inverse Document Frequency’ (TF-IDF) (Grimmer and Stewart, 2013; Altaweel *et al.*, 2019), we extracted from each prefecture-level policy document five keywords whose importance was proportional to the number of times they appeared in the document and was inversely proportional to their frequency of appearance in other documents in the policy database. The nature of policy actions for each document was assessed through these policy keywords. We further identified 4223 prefecture-level UEBMI policies, which had been defined as policies with the Chinese character referring to ‘UEBMI’ in the title or one of the extracted keywords.

In addition, we drew individual-level data from the longitudinal China Health and Nutrition Survey (CHNS). Cofounded by the Chinese Center for Disease Control and Prevention and the University of North Carolina at Chapel Hill, the CHNS was conducted in 52 prefectures in 1989, 1991, 1993, 1997, 2000, 2004, 2006, 2009, 2011 and 2015. It used a stratified cluster sampling strategy to collect data related to participants’ health spending, health services utilization, nutrition and income. In this study, we used the CHNS data from seven waves, beginning with the wave from 1997—the year before the central government formally enacted the UEBMI—and the six waves after that (2000–2015). Furthermore, we analysed data for the working population, defined as individuals who had formal jobs, because they are the primary target groups of the UEMBI. The observations of employees who participated in other SHI programs were excluded. The final sample included 13 488 individual observations, with 5938 UEBMI enrollees and 7550 uninsured workers. These observations were made up of 7778 unique individuals with on average 1.734 observations per participant across the seven selected waves between 1997 and 2015. Among these individuals, 4451 participated in the CHNS only once, while the rest participated in the survey at least twice. Specific individual-level data processing is presented in Supplementary Panel A. We subsequently matched prefecture-level policy data with individual-level survey data based on time and prefecture, yielding an unbalanced panel of 13 488 observations in 49 prefectures from the waves of 1997 through 2015.

### Variables

#### UEBMI predictors

To capture the orientation of UEBMI reform efforts at the prefectural level, we used the five automatically extracted policy keywords for each document. First, we manually categorized all policy keywords extracted from the prefectural UEBMI policies to one of the two categories presented in Table 1 and then assigned keywords that indicated either the benefit-expansion strategy or the cost-containment strategy to the relevant category. Moreover, we matched some semantically neutral keywords (e.g. reimbursement rate) with a few collocation words (e.g. enhance, improve, increase and expand) to explicitly demonstrate the reform orientation. Supplementary Table A presents those policy keywords and collocation items in details. Supplementary Panel B exhibits an example of how the semantically neutral keywords were handled.

Second, in each policy document, we counted all identified keywords allocated to the two policy categories and then aggregated the frequencies of the categorized keywords at the prefectural level. For example, the administration of Guangzhou issued three UEBMI policy documents in 2012, one of which included the keywords (and collocation items) ‘increasing reimbursement rates’ (frequency: 10), ‘supplementary programs’ (4) and ‘per diem payment’ (2). Each of the other two documents included the keyword ‘management of designated health facilities’ (three each). The frequency of the benefit-expansion keyword was 14 (= 10 + 4), whereas the frequency of the cost-containment keyword was 8 (= 2 + 3 + 3) for Guangzhou in 2012. To account for the long-term influence of UEBMI policies, we used yearly cumulative frequency of aggregated keywords from the year of UEBMI’s launch to the year of the observation for a prefecture, and eventually we were able to construct two prefecture-level measures: ‘benefit-expansion reforms’ and ‘cost-containment reforms’. We further integrated these prefecture-level measures into individual-level survey data as noted above.

Furthermore, in order to examine the impacts of UEBMI enrolment on OOP spending, we constructed a micro measure of ‘individual enrolment in the UEBMI’, which was represented by a dichotomous variable indicating whether an individual was enrolled in the UEBMI or was uninsured in the year of the survey.

#### Outcome variable

The CHNS provided information on OOP spending for an individual’s medical treatment, self-treatment and preventive behaviours during the 4 weeks prior to each survey wave. We adjusted the OOP spending data from all survey waves to 2015 levels, using the price inflation index, and then took the natural logarithm of OOP spending to ensure that this variable was close to normal distribution.

#### Control variables

China’s health insurance reforms have coincided with multiple supply-side reforms seeking to streamline health service delivery and regulating pharmaceutical markets (Liu *et al.*, 2017; Yip *et al.*, 2019; He et al., 2022). To control for the impacts of supply-side reforms on individual OOP spending, we took advantage of our health policy database to construct two supply-side variables: ‘service delivery reforms’ and ‘pharmaceutical reforms’. We extracted keywords from all prefecture-level health policy documents, assigned the identified keywords to the service delivery and pharmaceutical categories and then aggregated them at the prefectural level. Details of these keywords and their classifications are presented in Supplementary Table A.

Several individual-level covariates were controlled for, including days of inability to perform normal activities due to illness, severity of illness, chronic conditions, health services utilization, age, years of schooling, household size and the natural logarithm of per capita household income. Specific measurements and summary statistics for all variables are shown in Tables 2 and 3, respectively.

**Table 2.** Variables and measurements

Variable	Measurement
‘Outcome variable’	
Log (OOP spending)	The natural logarithm of individual OOP health spending during the 4 weeks prior to the survey. We added the value of 1 to all OOP spending data to avoid missingness when performing the logarithm calculation
‘UEBMI predictors’	
Benefit-expansion reforms	Yearly cumulative frequency of policy keywords aiming to expand UEBMI benefit package among texts of policies enacted by a prefecture
Cost-containment reforms	Yearly cumulative frequency of policy keywords aiming to achieve cost containment for the UEBMI among texts of policies enacted by a prefecture
UEBMI enrolment	A dummy variable indicating whether or not an individual enrolled in the UEBMI in a given survey year (0 = uninsured and 1 = enrolled in the UEBMI)
‘Supply-side covariates’	
Service delivery reforms	Yearly cumulative frequency of policy keywords aiming to improve the efficiency of health service delivery among texts of policies enacted by a prefecture since 1997
Pharmaceutical reforms	Yearly cumulative frequency of policy keywords aiming to regulate pharmaceutical markets among texts of policies enacted by a prefecture since 1997
‘Individual-level covariates’	
Days of inability to perform normal activities due to illness	Days unable to perform normal activities owing to an illness during the 4 weeks prior to the survey
Severity of illness	The severity of the illnesses that an individual had during the 4 weeks prior to the survey, including four categories (0 = no illness, 1 = not severe, 2 = somewhat severe and 3 = very severe)
Chronic conditions	A dummy variable indicating whether or not an individual had chronic diseases
Health services utilization	A dummy variable indicating whether or not an individual utilized outpatient or inpatient services in the 4 weeks prior to the survey
Age	An individual’s age in years
Schooling years	Schooling years of an individual
Household size	The number of the members in a household
Log (per capita household income)	The natural logarithm of per capita income of a household during the year prior to the survey

**Table 3.** Summary statistics of variables

Variable	*N*	Mean	SD
‘Outcome variable’			
Log (OOP spending)	13 433	0.554	1.714
‘UEBMI predictors’			
Benefit-expansion reforms	13 231	6.052	14.160
Cost-containment reforms	13 231	1.301	5.406
UEBMI enrolment	13 488	0.440	0.496
‘Supply-side covariates’			
Service delivery reforms	13 231	107.892	253.430
Pharmaceutical reforms	13 231	33.790	94.037
‘Individual-level covariates’			
Days of inability to perform normal activities due to illness	13 488	0.283	2.250
Severity of illness: no illness	13 488	0.856	0.352
Severity of illness: not severe	13 488	0.059	0.236
Severity of illness: somewhat severe	13 488	0.071	0.257
Severity of illness: very severe	13 488	0.014	0.118
Chronic conditions	13 488	0.042	0.201
Health services utilization	13 488	0.079	0.269
Age	13 486	43.195	15.793
Schooling years	13 345	9.503	3.551
Household size	13 427	3.701	1.510
Log (per capita household income)	13 427	8.974	1.372

### Empirical strategies

First, to estimate the macro-level impacts of prefectural UEBMI policy on individual OOP spending, we used a two-way fixed-effects model and controlled for year and individual fixed effects. The logarithm of OOP spending of UEBMI enrollees and uninsured populations was regressed on the two policy variables (‘benefit-expansion reforms’ and ‘cost-containment reforms’). The two continuous variables that indicated the adoption of benefit-expansion and cost-containment reforms were set to zero when a prefecture did not implement the UEBMI, and they had diverse values across prefectures and survey years after local adoptions of the UEBMI, with higher values reflecting higher levels of exposure to relevant reforms. Moreover, an interaction analysis was used to examine the interaction effects of the categories of policies on OOP spending. The equations used for those tests were set as follows:

(1.1) $$\eqalign{\ln \left( {OOPS_{ijt}^G} \right) = & \beta _0^G + \beta _1^Gexpansio{n_{jt}} + \beta _2^Gcontainmen{t_{jt}} \cr & + {\lambda ^G}Z_{jt}^G + {\delta ^G}X_{ijt}^G + Y_t^G + I_i^G + \varepsilon _{ijt}^G}$$

(1.2) $$\eqalign{\ln \left( {OOPS_{ijt}^G} \right) = & \beta _0^G + \beta _1^Gexpansio{n_{jt}} + \beta _2^Gcontainmen{t_{jt}} \cr & + \beta _3^Gexpansio{n_{jt}}*containmen{t_{jt}} + {\lambda ^G}Z_{jt}^G \cr & + {\delta ^G}X_{ijt}^G + Y_t^G + I_i^G + \varepsilon _{ijt}^G}$$

where G = {*A, E, U*}, and *A,*$E,$*and*$U$ represent all individuals, UEBMI enrollees and uninsured individuals, respectively. Subscripts $i$, $j$and $t$index the individual, prefecture and survey year, respectively. The $\ln \left( {OOPS_{ijt}^G} \right)$is the natural logarithm of OOP spending. The main predictors $expansio{n_{jt}}$ and $containmen{t_{jt}}$ are the prefecture- and year-specific cumulative word frequencies of the benefit-expansion and cost-containment reforms, respectively. The expression $expansio{n_{jt}}{\rm{*}}containmen{t_{jt}}$ is an interaction item of the two reforms. The term $Z_{jt}^G$ is a vector of supply-side covariates, and $X_{ijt}^G$ is a vector of individual-level covariates. The terms $Y_t^G$ and $I_i^G$ account for year fixed effects and individual fixed effects, respectively.

Second, the micro-level impacts of individuals’ UEBMI enrolment on their OOP spending were examined. Although UEBMI is legally mandatory for workers with formal contract, its enforcement in the initial stage was not strictly by-the-book, but was influenced by many other factors such as local economic growth, government fiscal and administrative capacity, labour power and formality of the industry sector (Liu, 2011). When examining the impact of insurance enrolment on health spending, some of these elements that affect both UEBMI enrolment and health spending may serve as confounders, leading to an endogeneity problem. To address that potential problem, we adopted an instrumental variable (IV) method and constructed two instruments: prefecture-level UEBMI penetration and GMI penetration, defined as the percentage of enrollees in relevant insurance programs in all working populations of a prefecture. The prefecture-level UEBMI penetration can affect a worker’s enrolment in the program through either a peer-effect mechanism (i.e. a worker may be motivated to enrol by the prevalence of enrolment among his or her counterparts in the workplace, if UEBMI participation is not strictly enforced in a prefecture) or a policy-effect mechanism (i.e. the UEBMI penetration captures the government’s efforts to expand UEBMI coverage). We chose GMI penetration as the instrument because the Chinese government has been working hard to replace GMI coverage with UEBMI coverage since the launch of the UEBMI. If GMI coverage is high in a prefecture, the likelihood of a worker enrolling in the UEBMI is reduced.

Using a three-way interaction analysis of UEBMI enrolment, benefit-expansion reforms and cost-containment reforms, we investigated the heterogeneous effects of UEBMI enrolment on OOP spending under different prefectural UEBMI policy conditions. The equations for these estimations were set as follows:

(2.1) $$ \eqalign{\ln \left( {OOP{S_{ijt}}} \right) = \ & {\beta _0} + {\beta _1}UEBMIenrol{l_{ijt}} \cr & + \lambda {Z_{jt}} + \delta {X_{ijt}} + {Y_t} + {I_i} + {\varepsilon _{ijt}}}$$

(2.2) $$ \eqalign{\ln \left( {OOP{S_{ijt}}} \right) = \ & {\beta _0} + {\beta _1}UEBMIenrol{l_{ijt}} + {\beta _2}expansio{n_{jt}} \cr & + {\beta _3}containmen{t_{jt}} + {\beta _4}UEBMIenrol{l_{ijt}}\cr & {\rm{*}} expansio{n_{jt}} + {\beta _5}UEBMIenrol{l_{ijt}}\cr & {\rm{*}}containmen{t_{jt}} + {\beta _6}expansio{n_{jt}}\cr & {\rm{*}}containmen{t_{jt}} + {\beta _7}UEBMIenrol{l_{ijt}}\cr & {\rm{*}}expansio{n_{jt}}{\rm{*}}containmen{t_{jt}} \cr & + \lambda {Z_{jt}} + \delta {X_{ijt}} + {Y_t} + {I_i} + {\varepsilon _{ijt}}} $$

where $UEBMIenrol{l_{ijt}}$ denotes an individual’s enrolment status in the UEBMI or the probability of the person’s enrolment predicted by UEBMI penetration, GMI penetration and other covariates. The expressions $UEBMIenrol{l_{ijt}}$${\rm{*}}expansio{n_{jt}}$ and $UEBMIenrol{l_{ijt}}{\rm{*}}containmen{t_{jt}}$ are the interaction items of UEBMI enrolment and benefit-expansion reforms and of UEBMI enrolment and cost-containment reforms, respectively. The expression $UEBMIenrol{l_{ijt}}$${\rm{*}}expansio{n_{jt}}{\rm{*}}containmen{t_{jt}}$ is the three-way interaction item of UEBMI enrolment and the two reforms.

## Results

### The subnational landscape of UEBMI policy

**Figure 1.**
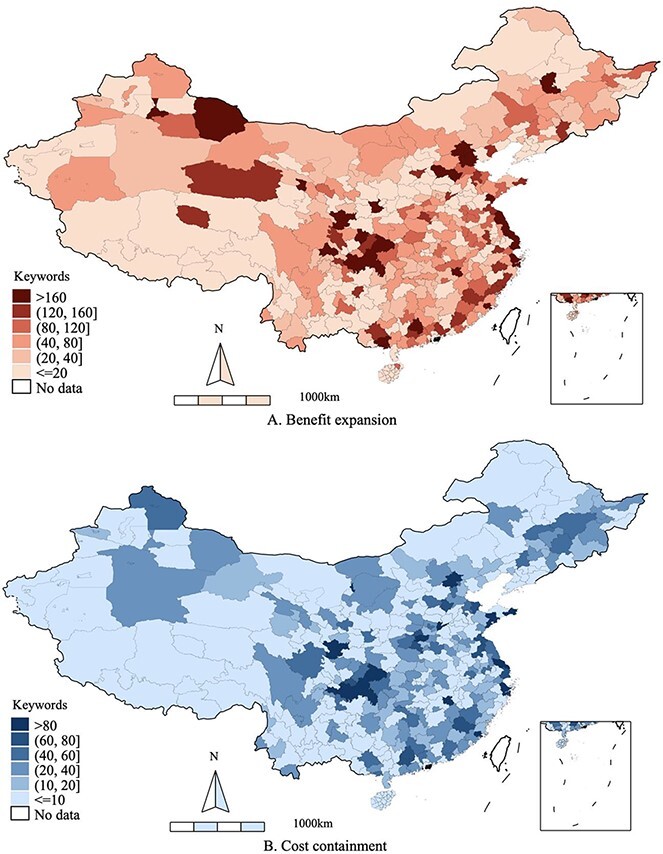
Local policy discrepancy in the UEBMI reforms

Notes: Benefit expansion denotes the cumulative number of policy keywords aiming to expand the UEBMI’s benefit packages among texts of policies enacted between the year of introducing the UEBMI and 2015. Cost containment denotes the cumulative number of policy keywords aiming to achieve cost containment for the UEBMI among texts of policies enacted between the year of introducing the UEBMI and 2015.

Figure 1 depicts the geographic variations in UEBMI’s benefit-expansion reforms and cost-containment reforms. Apparently, there were substantial policy disparities among prefectures with regard to both types of reforms. Many prefectures in Eastern China (e.g. Beijing, Nantong, Ningbo and Putian) and some prefectures in the Western regions (e.g. Chongqing and Longnan) promulgated numerous policies that targeted the expansion of the UEBMI’s benefit packages as well as cost containment. In these prefectures, the cumulative word frequencies for the benefit-expansion and cost-containment reforms from the year of the prefecture’s UEBMI introduction to 2015 amounted to more than 160 and 80, respectively. In contrast, one-third of the prefectures (111 out of 333) had cumulative word frequencies of less than 20 for both types of reforms. The results also indicate that benefit-expansion reforms were given a higher priority by most prefectures. In 2015, 84 prefectures had a cumulative word frequency of more than 80 for benefit-expansion reforms, compared with only 14 prefectures with a high word frequency for cost-containment reforms.

### Macro-level impacts of UEBMI policy reforms on OOP spending

**Figure 2.**
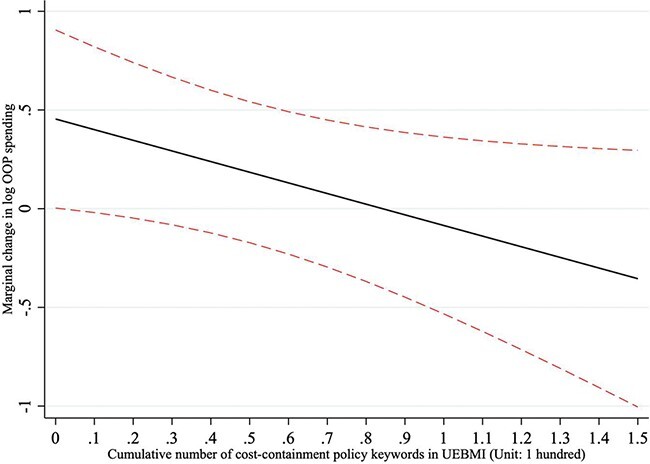
The marginal effects of benefit-expansion reforms on OOP spending of all individuals at various levels of cost-containment reforms

Notes: The dark solid line indicates the marginal effects of yearly cumulative frequency of keywords denoting benefit-expansion reforms on the logarithm of OOP spending at representative values of cumulative frequency of keywords denoting cost-containment reforms. The light dotted lines indicate 95% confidence intervals.

Table 4 reports the regression results for the impacts of prefecture-level UEBMI policy reforms on OOP spending of all individuals, UEBMI enrollees and the uninsured population, respectively. For all individuals, neither benefit-expansion reforms nor cost-containment reforms at the prefectural level had a significant impact on OOP spending (Model 1). We conducted an interaction analysis in Model 2. The interaction effect of both types of policies on reducing OOP spending was statistically significant (Benefit-expansion × Cost-containment = −0.546, *P* < 0.10). In prefectures that had adopted only benefit-expansion policies but no cost-containment efforts, the UEBMI policy significantly increased OOP spending (Benefit-expansion = 0.473, *P* < 0.05, if keyword frequency for cost-containment reforms = 0). Figure 2 presents the marginal effects of prefectural benefit-expansion reforms on OOP spending of all individuals at various levels of cost-containment reforms. As prefectures implemented increasing cost-containment strategies, the positive relationship between benefit-expansion reforms and OOP spending gradually weakened.

**Table 4.** Estimated effects of the UEBMI benefit-expansion and cost-containment reforms on OOP spending

	All individuals		UEBMI enrollees		Uninsured workers	
Outcome: Log (OOP spending)	Model 1	Model 2	Model 3	Model 4	Model 5	Model 6
‘UEBMI policy predictors’						
Benefit-expansion reforms	0.319 (0.201)	0.473** (0.230)	0.318 (0.333)	0.609* (0.315)	−0.040 (0.374)	0.227 (0.477)
Cost-containment reforms	−0.248 (0.406)	0.108 (0.433)	−0.422 (0.545)	0.350 (0.557)	0.876 (0.828)	1.695*** (0.538)
Benefit-expansion × Cost-containment		−0.546* (0.279)		−1.395*** (0.319)		−0.589 (0.431)
‘Supply-side covariates’						
Service delivery reforms	0.080*** (0.021)	0.076*** (0.021)	0.096*** (0.027)	0.091*** (0.026)	0.045 (0.057)	0.031 (0.062)
Pharmaceutical reforms	−0.165*** (0.057)	−0.158*** (0.057)	−0.203*** (0.071)	−0.192*** (0.070)	−0.040 (0.073)	−0.029 (0.077)
‘Individual-level covariates’						
Days of inability to perform normal activities due to illness	0.053*** (0.018)	0.053*** (0.018)	0.062*** (0.023)	0.063*** (0.023)	0.043 (0.036)	0.043 (0.036)
Severity of illness	1.135*** (0.066)	1.133*** (0.066)	1.056*** (0.082)	1.051*** (0.082)	1.276*** (0.128)	1.275*** (0.128)
Chronic conditions	0.086 (0.159)	0.087 (0.159)	0.035 (0.203)	0.039 (0.203)	0.392 (0.331)	0.391 (0.331)
Health services utilization	2.554*** (0.156)	2.558*** (0.156)	2.538*** (0.202)	2.546*** (0.202)	2.383*** (0.252)	2.388*** (0.252)
Age	0.045 (0.069)	0.043 (0.069)	0.069 (0.136)	0.064 (0.137)	−0.004 (0.079)	−0.006 (0.078)
Schooling years	−0.009 (0.013)	−0.009 (0.013)	−0.011 (0.021)	−0.010 (0.021)	−0.001 (0.014)	−0.000 (0.014)
Household size	0.001 (0.022)	0.001 (0.022)	0.029 (0.050)	0.028 (0.050)	0.013 (0.019)	0.013 (0.019)
Log (per capita household income)	0.021 (0.014)	0.021 (0.014)	0.034 (0.057)	0.034 (0.057)	0.011 (0.010)	0.011 (0.010)
Constant	−1.667 (2.427)	−1.626 (2.430)	−4.305 (7.799)	−4.113 (7.821)	0.009 (2.503)	0.063 (2.491)
Individual fixed effects	Y	Y	Y	Y	Y	Y
Year fixed effects	Y	Y	Y	Y	Y	Y
*R* ^2^	0.567	0.567	0.502	0.502	0.690	0.690
Observations	13 033	13 033	5822	5822	7211	7211

Notes: *P < 0.10,

** *P* < 0.05,

*** *P* < 0.01.

Robust standard errors in parentheses. To obtain appropriate coefficients in regression analyses, we used 100 of keyword frequency as the unit of the variables of benefit-expansion reforms, cost-containment reforms, service delivery reforms and pharmaceutical reforms, respectively.

**Figure 3.**
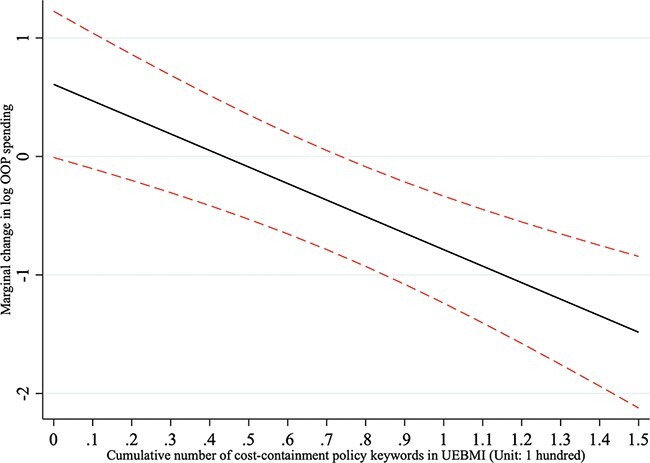
The marginal effects of benefit-expansion reforms on OOP spending of UEBMI enrollees at various levels of cost-containment reforms

Notes: The dark solid line indicates the marginal effects of yearly cumulative frequency of keywords denoting benefit-expansion reforms on the logarithm of OOP spending at representative values of cumulative frequency of keywords denoting cost-containment reforms. The light dotted lines indicate 95% confidence intervals.

For UEBMI enrollees, prefectural benefit-expansion reforms increased OOP spending, whereas cost-containment reforms decreased it. However, neither relationship was statistically significant at the 0.10 level, as Model 3 demonstrates. Interestingly, Model 4 reveals that the interaction effect of the two policy measures on lowering OOP spending was statistically significant (Benefit-expansion × Cost-containment = −1.395, *P* < 0.01). However, benefit-expansion reforms predicted a significant increase in OOP spending in the prefectures where there were no cost-containment policies (Benefit-expansion reforms = 0.609, *P* < 0.10, if keyword frequency for cost-containment reforms = 0). Figure 3 depicts the marginal effects of prefectural benefit-expansion reforms on OOP spending of UEBMI enrollees at various levels of cost-containment reforms. In prefectures that had adopted a large number of cost-containment policies (keyword frequency > 44), benefit-expansion reforms predicted a significant decrease in OOP spending.

For uninsured individuals, neither type of reforms at the prefectural level had a significant effect on their OOP spending (Model 5). When we performed an interaction analysis in Model 6, cost-containment reforms in the prefectures that had adopted no benefit-expansion policies actually predicted a significant increase in OOP spending (Cost-containment = 1.695, *P* < 0.01, if keyword frequency for benefit-expansion reforms = 0), thus implying that in those prefectures, the UEBMI cost-containment reforms had an unintended spillover effect of additional costs to the uninsured.

### Micro-level impacts of UEBMI enrolment on OOP spending

We investigated micro-level impacts of UEBMI on individual OOP spending using the micro measure of UEBMI enrolment. Before the potential problem of endogeneity was fully addressed, UEBMI enrolment had no significant relationship with OOP spending, as shown in Model 7 of Table 5. Similarly, UEBMI enrolment yielded no significant impact on OOP spending when an IV analysis was performed in Model 8. Moreover, there was no evidence of endogeneity in the relationship between UEBMI enrolment and OOP spending in Model 8 (*P* for the endogeneity test = 0.303).

**Table 5.** Estimated effects of UEBMI enrolment on OOP spending

	UEBMI enrolment		UEBMI enrolment × Policy reforms
Outcome: Log (OOP spending)	Model 7	Model 8 (IV)	Model 9
‘UEBMI predictors’			
UEBMI enrolment	0.007 (0.053)	0.169 (0.163)	0.012 (0.057)
Benefit-expansion reforms			0.078 (0.438)
Cost-containment reforms			1.674*** (0.578)
UEBMI enrolment × Benefit-expansion			0.439 (0.443)
UEBMI enrolment × Cost-containment			−1.595** (0.655)
Benefit-expansion × Cost-containment			−0.526 (0.331)
UEBMI enrolment × Benefit-expansion × Cost-containment			−0.261 (0.342)
UEBMI enrolment + UEBMI enrolment × Benefit-expansion			0.451 (0.424)
UEBMI enrolment + UEBMI enrolment × Cost-containment			−1.583** (0.659)
UEBMI enrolment + UEBMI enrolment × Benefit-expansion + UEBMI enrolment × Cost-containment + UEBMI enrolment × Benefit-expansion × Cost-containment			−1.406*** (0.445)
‘Supply-side covariates’			
Service delivery reforms	0.080*** (0.022)	0.078*** (0.022)	0.077*** (0.022)
Pharmaceutical reforms	−0.171*** (0.056)	−0.165*** (0.055)	−0.160*** (0.057)
‘Individual-level covariates’			
Days of inability to perform normal activities due to illness	0.053*** (0.018)	0.053*** (0.018)	0.053*** (0.018)
Severity of illness	1.135*** (0.066)	1.131*** (0.065)	1.133*** (0.066)
Chronic conditions	0.085 (0.159)	0.089 (0.157)	0.088 (0.159)
Health services utilization	2.553*** (0.156)	2.561*** (0.151)	2.559*** (0.156)
Age	0.047 (0.069)	0.047 (0.072)	0.044 (0.069)
Schooling years	−0.009 (0.013)	−0.010 (0.013)	−0.009 (0.013)
Household size	0.002 (0.022)	0.003 (0.022)	0.000 (0.022)
Log (per capita household income)	0.021 (0.014)	0.019 (0.014)	0.021 (0.014)
Constant	−1.754 (2.421)	−1.841 (2.421)	−1.641 (2.433)
Individual fixed effects	Y	Y	Y
Year fixed effects	Y	Y	Y
Weak instrument (*F*-statistic)		330.468	
Overidentification (*P*-value)		0.578	
Endogeneity (*P*-value)		0.303	
*R* ^2^	0.567	0.566	0.568
Observations	13 033	13 033	13 033

Notes: *P < 0.10,

** *P* < 0.05,

*** *P* < 0.01.

Robust standard errors in parentheses. ‘IV’ stands for instrumental variable. We adopted the IV method in Model 8. As shown in Model 8, the predictor of UEBMI enrolment used in the regression estimation is not endogenous (*P*-value for endogeneity test is 0.303). Therefore, we did not use the IV method when interacting UEBMI enrolment and policy reforms in Model 9. Using the linear combination technique, we conducted a set of post hoc analyses to estimate the regression coefficients and their statistical significance for a set of aggregated variables, including ‘UEBMI enrolment + UEBMI enrolment × Benefit-expansion’, ‘UEBMI enrolment + UEBMI enrolment × Cost-containment’ and ‘UEBMI enrolment + UEBMI enrolment × Benefit-expansion + UEBMI enrolment × Cost-containment + UEBMI enrolment × Benefit-expansion × Cost-containment’. To obtain appropriate coefficients in regression analyses, we used 100 of keyword frequency as the unit of the variables of benefit-expansion reforms, cost-containment reforms, service delivery reforms and pharmaceutical reforms, respectively.

We further estimated the moderating role that prefecture-level reforms played in the relationship between UEBMI enrolment and OOP spending. Model 9 suggests that in prefectures where ‘only’ benefit-expansion policies were promulgated, UEBMI enrolment registered no significant impact on OOP spending (UEBMI enrolment  + UEBMI enrolment × Benefit-expansion = 0.451, *P* = 0.288). However, in prefectures that enacted cost-containment policies ‘only’, UEBMI enrolment significantly reduced OOP spending (UEBMI enrolment + UEBMI enrolment × Cost-containment = −1.583, *P* < 0.05). Nevertheless, uninsured workers’ OOP spending increased significantly in those prefectures with only cost-containment policies (Cost-containment = −1.595, *P* < 0.01). In prefectures where both categories of reforms had been rolled out, UEBMI enrolment significantly decreased individuals’ OOP spending (UEBMI enrolment + UEBMI enrolment × Benefit-expansion + UEBMI enrolment × Cost-containment + UEBMI enrolment × Benefit-expansion × Cost-containment = −1.406, *P* < 0.01). Meanwhile, the UEBMI policy in such prefectures generated no spillover effect onto uninsured workers’ OOP spending (Benefit-expansion × Cost-containment = −0.526, *P* = 0.378).

**Figure 4.**
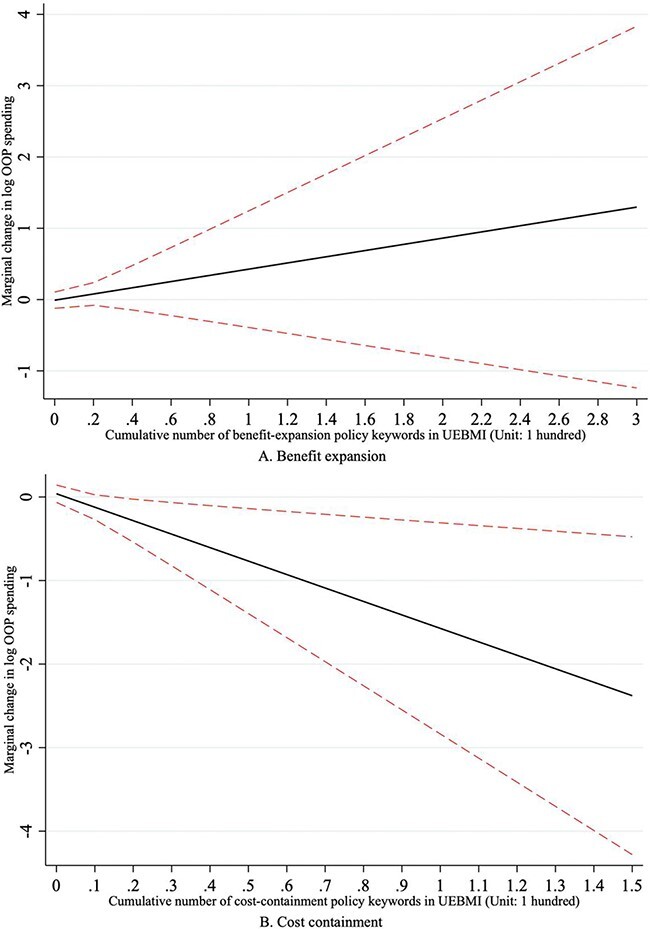
The marginal effects of UEBMI enrolment on OOP spending at various levels of benefit-expansion reforms and cost-containment reforms, respectively

Notes: The dark solid lines represent the marginal effects of UEBMI enrolment on the logarithm of OOP spending at representative values of yearly cumulative frequency of keywords denoting benefit-expansion reforms (Figure 3A) and cost-containment reforms (Figure 3B), respectively. When presenting the marginal effects of UEBMI enrolment on OOP spending at various levels of benefit-expansion reforms, we controlled for the level of cost-containment reforms (and other covariates) and vice versa.

Figure 4 plots the marginal effects of UEBMI enrolment on OOP spending at different levels of reforms. Enrolment in the UEBMI had no significant impacts on OOP spending across prefectures that deployed different levels of benefit-expansion reforms. The OOP spending reduction as a result of UEBMI enrolment became obvious in prefectures that implemented a large number of cost-containment policies.

### Robustness tests

We conducted a two-part model (2PM) analysis to test the robustness of the main results. At a fixed point of time, most people normally do not have illness or prefer self-treatment in case of very minor illness, and therefore, a survey would record zero value for OOP spending. As a result, healthcare spending data reported in most surveys do not conform to normal distribution. Under such circumstances, previous studies typically employed the 2PM method, assuming that seeking treatment and the decision of spending are independent and discrete decisions for individuals (Buntin and Zaslavsky, 2004; O’Donnell *et al.*, 2008). In this study, we used a 2PM consisting of a probit regression of the probability of incurring any OOP spending (i.e. OOP spending = 0 or not) and a linear regression of positive OOP spending with log link (i.e. the natural logarithm of OOP spending if OOP spending > 0).

As shown in Supplementary Table B, the 2PM results corroborate our main results. Conditional on having OOP spending, an individual’s OOP spending decreased when both categories of policies were adopted, but increased when only benefit-expansion reforms were implemented. Supplementary Table C presents the 2PM results for the impacts of UEBMI enrolment on OOP spending. Conditional on having OOP spending, UEBMI enrolment exerted no significant effect on OOP spending in prefectures that adopted only benefit-expansion reforms. UEBMI enrolment reduced OOP spending in prefectures that adopted both types of policies; however, the relationship was not statistically significant at the 0.10 level. Uninsured workers’ OOP spending increased in prefectures that adopted only cost-containment reforms, which was neither statistically significant.

## Discussion and conclusion

Analysing a novel health policy database in China, this study developed a text-mining approach for measuring health policies that have subnational disparities and then applied that approach to a case study examining the impacts of prefectural UEBMI policies on individuals’ OOP spending. Three key findings emerged. First, the UEBMI was associated with substantial disparities among prefectures in terms of reform orientations. Second, the macro-level UEBMI policy had considerably lowered enrollees’ OOP spending in prefectures that prioritized the synergy between benefit-expansion and cost-containment reforms. In contrast, without such synergic efforts, prefectures that had implemented benefit-expansion reforms only witnessed a significant increase in enrollees’ spending, whereas prefectures that had adopted only cost-containment reforms observed a significant increase in uninsured workers’ OOP spending. Third, the micro-level impacts of UEBMI enrolment on OOP spending were conditional on how benefit-expansion and cost-containment reforms interacted at the macro level. Prefectures that promoted the synergy between the two types of reforms received the desired policy outcomes: individuals enrolled in the UEBMI in such prefectures benefited from reduced OOP spending. In contrast, prefectures that had implemented poorly coordinated reforms encountered problematic policy consequences: UEBMI enrolment exerted no significant effect on OOP spending, under the condition that only benefit-expansion reforms were in force in a prefecture. Prefectures that had adopted only cost-containment reforms actually experienced an increase in OOP spending by uninsured workers.

These findings suggest that investigations of policy effects in decentralized health systems should pay adequate attention to local variations of the same national policy. The existing policy evaluation literature has largely not been able to capture this subnational policy heterogeneity. In order to gain a more nuanced yet thorough understanding, a rigorous analytic protocol should be built upon a comprehensive measurement of local policy interventions. In this vein, contextualizing policy measurements could help researchers extract comprehensive measures to capture diverse policy efforts across regions. When measuring health policies, researchers could synthesize policy options and specifics adopted by the relevant authorities through mining policy texts. Indeed, the emergence of policy big-data techniques has enabled researchers to do so. Our study represents one such attempt.

Furthermore, evaluation studies that utilize simplified policy measures could be improved by considering policy disparities at the subnational level. Integrating subnational policy analysis into a micro-level impact analysis could help researchers identify diverse pathways through which a policy produces effects. Such insights are of high value for both researchers and policymakers to better unpack the complexities of the policy process and further improve policy interventions. In this study, analyses based solely on individual insurance enrolment suggested that UEBMI generated no significant impact on OOP spending—a finding that could lead to the conclusion that the SHI program is inefficient in financial protection. However, policymakers may be perplexed as to the exact mechanisms through which such inefficiency was produced. Was it due to a low-level risk-pooling mechanism? Alternatively, was it related to ill-functioning cost-containment mechanisms? By evaluating the interactions of individual UEBMI enrolment and prefectural policy reforms, we were able to provide more accurate policy implications: promoting synergy between benefit-expansion reforms and cost-containment reforms. Relying solely on either of those two types of reforms not only appears to undermine intended outcomes, but it can actually create adverse effects at the individual level. Only when the two reforms are synchronized can the UEBMI reduce OOP spending of enrollees and avoid a cost-shifting spillover effect on the uninsured.

To decompose the conundrum of intertwined policy mechanisms, future studies could employ automated text-mining tools to classify multiple interventions present in the same policy framework. In addition to the TF-IDF method used in this study, researchers could use supervised (e.g. naive Bayes, support vector machines, decision trees and *K*-nearest neighbour) and unsupervised (e.g. *K*-means, topic modelling and deep, convolutional or recurrent neural network) machine-learning techniques to extract and classify sophisticated policy efforts in policy texts (Grimmer and Stewart, 2013). Such techniques greatly empower researchers to explore how various interventions interact and determine policy outcomes.

In light of extensive local policy disparities, the Chinese policymakers have considered a range of measures to enhance central-local policy coherence and mitigate subnational policy variations. In 2018, the government attempted to address this issue by establishing the National Healthcare Security Administration. The new agency carried out a set of nationally unified rules such as new provider payment methods, reforming individual accounts and revamping the distorted drug pricing system. These reforms manifest a robust trend towards stronger government stewardship in healthcare (He et al., 2022). A key message directly arising from our study is that greater efforts should be paid to coordinate various policy interventions at the local level and pay due attention to their interactive effects, in order to maximize the overall impact of healthcare reforms.

This study is certainly not without limitations. First, some local governments may not disclose into the public domain all of the policy documents that they have issued, and therefore our access to policy big data may have been limited. Second, our text-mining approach may have been subject to measurement errors, because policy keywords may not accurately reflect the full intentions of relevant policy reforms. Third, our Occam’s Razor categorization of UEBMI reforms may not allow us to generalize the findings to every possible policy intervention. We strive to address these limitations in our future research by experimenting more-sophisticated text-mining tools.

## Supplementary Material

czac086_Supp

## Data Availability

This study is associated with a large corpus of health policy documents. The authors welcome request for access.

## Reflexivity statement

The research team is a dynamic one consisting of two mid-career associate professors and a doctoral student. Both genders are represented. KL and WTL are based in Mainland China while AJH is based in Hong Kong SAR. Team members complement each other very well in terms of research expertise. We are very proud that a pre-career doctoral student is able to participate in this project and make considerable contribution.
