# Supplementary material for: Evaluating health policies with subnational disparities: a text-mining analysis of the Urban Employee Basic Medical Insurance Scheme in China
Source: Health Policy Plan. 2022 Oct 11;38(1):83–96. doi: 10.1093/heapol/czac086 (PMC9849718; doi:10.1093/heapol/czac086)
Supplement: czac086_Supp [file czac086_supp.zip › Supplementary.docx]

**Supplementary material**

**Panel A.** CHNS data processing

We drew individual-level data from the longitudinal China Health and Nutrition Survey (CHNS), which was conducted in China between 1989 and 2015 through a collaboration between the University of North Carolina at Chapel Hill and the Chinese Center for Disease Control and Prevention. Since 1989, stratified cluster sampling was used to implement a tracking survey in the eight provinces of Liaoning, Jiangsu, Shandong, Henan, Hubei, Hunan, Guangxi, and Guizhou. The remaining waves incrementally expanded the survey scope to cover four more provinces, including Heilongjiang in 1997, and Beijing, Shanghai, and Chongqing in 2011. To date, 10 survey waves have been conducted in 52 prefectures in the years 1989, 1991, 1993, 1997, 2000, 2004, 2006, 2009, 2011, and 2015. Overall, the survey asked participants for information on their health spending, healthcare utilization, nutrition, and other such issues at the individual level, while asking about issues such as income and spending at the household level.

This study used data from seven survey waves, including the wave from 1997 – the year before the central government formally enacted the UEBMI in 1998 – and the six waves after that (2000-2015). Moreover, we analyzed individual-level data for the working population, defined as those having formal jobs (professional, technical, administrative, skilled, non-skilled, and service work). We focused on this set of individuals since, according to the UEBMI’s rules of eligibility, they are the target of the program. For this purpose, we omitted 37,216 observations of farmers, students, unemployed individuals, homeworkers, and other non-working populations. We also excluded 43,916 observations of individuals who had insurance through a non-UEBMI program like the NCMS, URBMI, GMI, LI, or CMS. We further excluded 8,896 individual-level observations of participants in Beijing, Shanghai, and Chongqing, which have not implemented the CHNS until 2011. The final individual-level sample included 13,488 individual observations, with 5,938 UEBMI enrollees and 7,550 uninsured workers in 49 prefectures between 1997 and 2015.

**Panel B.** The method for addressing the semantically neutral keywords

Amongst all keywords that were automatically extracted from policy documents, some of them (e.g., reimbursement rate) were semantically neutral, which cannot explicitly denote the orientations of policy reforms. To address this issue, we matched these semantically neutral keywords with a few collocation words, so as to explicitly demonstrate UEBMI reform orientations. In a policy document, we required that a semantically neutral keyword and one of its collocation items existed in the same sentence (i.e., the text between two adjacent arbitrary punctuation marks).

For example, for a piece of text “…increase the reimbursement rate for inpatient services within the scope of the UEBMI policy, and prudently maintain the UEBMI’s maximum payments for outpatient services”, we counted “reimbursement rate” as a benefit-expansion keyword because it appears together with a collocation item “increase” in the first sentence. We did not include “maximum payments” as a keyword for this text because one of the collocation terms, such as “enhance”, “improve”, “increase”, and “expand” did not appear in the same sentence. Details about all semantically neutral keywords and their collocation words are shown in Supplementary Table A.

**Table A.** Keywords identified in the health policy database

| Category | Strategy of reform | Policy keyword | Collocation item |
| --- | --- | --- | --- |
| ***A. UEBMI policy reforms*** | | | |
| Benefit expansion reforms | Boosting coinsurance rates and maximum payments | Reimbursement rate, insurance payment rate, payments by pooling fund, ceiling, maximum payment | Increase, enhance, expand, improve, adjust, cover |
|  |  | Deductible, threshold of payment, out-of-pocket payment, individual burden of payment | Decrease, reduce, decline, cancel |
|  | Broadening the scope of medicine catalogs | Medicine catalogue, three catalogues, scope of payment by insurance fund, Type A drug, Type B drug, medical insurance drug; diagnosis and treatment item catalogue, diagnosis and treatment item scope, diagnosis and treatment item for basic medical insurance; service facility standard, service facility scope, service facility catalogue, medical equipment and supplies catalogue, inpatient service standard | Increase, enhance, expand, improve, include, adjust, cover |
|  | Adopting supplementary programs | Supplementary medical insurance, critical illness insurance, reimbursement for critical illnesses, medical assistance, inpatient service assistance, outpatient service assistance, illness aid, private insurance, commercial insurance |  |
| Cost containment reforms | Promoting prospective payment methods | Payment reform, payment method reform, global budget, fixed fee, Diagnosis-Related Groups, payment for single diseases, charging for single diseases, points method payment, points payment, Diagnosis-Intervention Packet, capitation, per diem, payment by service unit |  |
|  | Containing insurance fund expenses | Cost per admission, cost per hospitalization, cost per day, cost of drugs outside the insurance catalogue as a share of total cost, length of stay, day of hospitalization, hospitalization rate, re-hospitalization rate, the number of inpatient admissions, ratio of the number of admissions to that of patients, control of total amounts, balance of total amounts, management of total amounts | Medical insurance + reform, control, restrict, reduce, and decrease |
|  | Negotiating medicine prices | Drug price negotiation, negotiation mechanism, price of chronic disease drug, price of ant-cancer drugs, price negotiation for medical supplies | Medical insurance |
|  | Improving the capacity of UEBMI agencies to manage contracted health facilities | Health insurance designated institutions, designated pharmacy, designated hospital, management by insurance agency, service by insurance agency, capability of insurance agency |  |
| ***B. Supply-side reforms*** | | | |
| Service delivery reforms | Reinforcing referral system | Referral, graded diagnosis and treatment, initial diagnosis at primary care facilities |  |
|  | Reforming incentive structure for physicians | Doctor’s salary, doctor’s income, legal income, personnel and remuneration system, distributional incentive mechanism, remuneration distribution, motivation of medical staff | Increase, enhance, expand, adjust, cover, encourage, reform, improve |
|  |  | Autonomy in personnel management, personnel system reform, employment system (rather than tenure system), staffing reform |  |
|  | Adjusting price schedule | Cancel drug markup, zero drug markup |  |
|  |  | Health service price, diagnosis price, registration fee, labor value | Increase, enhance, expand, adjust, cover, encourage, reform, improve |
|  | Imposing administrative orders to contain healthcare costs | Supplier-induced demand, over-medication, over-medical test, over-treatment, unnecessary services, inpatient admission for common diseases, prescription of expensive drugs, pubic hospital reform, separation of medical services and drug prescription, break the link between drug prescription and hospital revenue, cost control, control of spending, cost management, drug cost as a share of total cost, drug revenue, cost of medical supplies as a share of total cost, medical test cost as a share of total cost | reform, control, restrict, reduce, and decrease |
|  | Standardizing and digitalizing information of health services | Clinical pathway, diagnosis and treatment standard; electronic medical record, information system construction, medical record system, information infrastructure, unified coding, unified number, coding management, unified standard, unified style, unified input, unified filing, unified data | Health service, healthcare |
|  | Improving healthcare safety and quality | Safety supervision, safety management, rule of safety, accident, malpractice, error, special inspection, dispute, conflict, doctor-patient relation, doctor-patient communication, patient compliant, mediation; quality control, quality management, quality evaluation, service quality, rule of quality, quality care, quality nursing, specialty construction, emergency environment, emergency facilities, service quality | Health service, healthcare |
|  |  | Physician qualification, licensure for physician, practitioner training, medical education, humanistic quality, professional quality, behavior discipline, ethics improvement, ethics construction standardized training, job training, technical qualification, talent tank, expert tank, performance assessment |  |
|  |  | Convenient payment, convenient service, treatment by appointment, registration by appointment, one-stop billing, instant billing, instant reimbursement, treatment first and payment later |  |
|  | Building modern hospital management system | Corporate governance, modern management, internal governance, governance structure, modern corporate management, performance management, budget implementation, comprehensive budget, budget management | Hospital |
| Pharmaceutical reforms | Establishing a National Essential Medicine System | Essential medicine, essential medicine catalogue, national essential medicine | increase, enhancement, expansion, improve, adjust, and cover |
|  | Regulating medicine supplies | Drug circulation, drug wholesale, two-invoice system, one-invoice system |  |
|  | Regulating medicine bidding and procurement | Centralized pharmaceutical bidding and procurement, procurement with explicit demand, legal procurement, online procurement, drug bidding, drug procurement, medical supplies procurement, medical supplies bidding, 4+7, bidding ally, procurement ally |  |
|  | Reforming drug pricing | Maximum ex-factory drug price, pharmaceutical price, retail drug prices, drug price inflation, maximum retail drug price, secondary bargaining of drug price | Reform, control, restrict, reduce, and decrease |
|  |  | Market mechanism, encouraging competition, elimination of government pricing | Drug |

Notes: Each keyword and one of its collocation items must exist in the same sentence in a policy document. If not, a keyword will not be deemed as a word denoting a specific orientation of reform.

**Table B.** Robustness tests of the effects of the UEBMI benefit-expansion and cost-containment reforms on OOP spending using the two-part models

|  | All individuals | | UEBMI enrollees | | Uninsured workers | |
| --- | --- | --- | --- | --- | --- | --- |
|  | Model 1 | Model 2 | Model 3 | Model 4 | Model 5 | Model 6 |
| *Part 1: occurrence of OOP spending as the outcome variable* | | |  |  |  |  |
| Benefit-expansion reforms | 0.189(0.184) | 0.317(0.212) | 0.189(0.178) | 0.260(0.206) | 0.095(0.542) | 0.724(0.596) |
| Cost-containment reforms | 0.537(0.451) | 0.844(0.525) | 0.506(0.424) | 0.670(0.488) | -0.097(1.277) | 5.095(3.962) |
| Benefit-expansion*Cost-containment |  | -0.370(0.243) |  | -0.214(0.240) |  | -16.968(14.690) |
| Service delivery reforms | 0.017(0.017) | 0.014(0.018) | 0.024(0.016) | 0.022(0.017) | -0.026(0.066) | -0.028(0.069) |
| Pharmaceutical reforms | -0.071(0.046) | -0.064(0.046) | -0.088^**^(0.043) | -0.085^*^(0.044) | 0.157(0.137) | 0.135(0.140) |
| Days of inability to perform normal activities due to illness | -0.033^***^(0.008) | -0.033^***^(0.008) | -0.025^***^(0.009) | -0.025^***^(0.009) | -0.049^***^(0.017) | -0.048^***^(0.017) |
| Severity of illness | 1.094^***^(0.034) | 1.093^***^(0.034) | 0.972^***^(0.036) | 0.971^***^(0.036) | 1.381^***^(0.080) | 1.376^***^(0.080) |
| Chronic conditions | -0.078(0.107) | -0.077(0.107) | -0.035(0.101) | -0.034(0.101) | 0.095(0.386) | 0.099(0.389) |
| Health services utilization | 1.403^***^(0.071) | 1.406^***^(0.071) | 1.233^***^(0.082) | 1.235^***^(0.082) | 1.700^***^(0.145) | 1.710^***^(0.145) |
| Age | -0.005^***^(0.002) | -0.005^**^(0.002) | -0.005^**^(0.002) | -0.005^**^(0.002) | -0.002(0.004) | -0.002(0.004) |
| Schooling years | -0.003(0.007) | -0.003(0.007) | -0.007(0.008) | -0.007(0.008) | 0.008(0.015) | 0.007(0.015) |
| Household size | -0.064^***^(0.018) | -0.063^***^(0.018) | -0.060^***^(0.023) | -0.060^***^(0.023) | -0.064^*^(0.034) | -0.065^*^(0.034) |
| Log (per capita household income) | 0.006(0.018) | 0.006(0.018) | 0.005(0.032) | 0.005(0.032) | 0.016(0.027) | 0.018(0.027) |
| Constant | -2.028^***^(0.214) | -2.035^***^(0.215) | -1.505^***^(0.373) | -1.528^***^(0.375) | -2.497^***^(0.364) | -2.500^***^(0.366) |
| Year fixed effects | Y | Y | Y | Y | Y | Y |
| Pseudo R^2^ | 0.574 | 0.574 | 0.492 | 0.492 | 0.683 | 0.684 |
| Observations | 13,033 | 13,033 | 5,822 | 5,822 | 7,211 | 7,211 |
| *Part 2: conditional OOP spending (in log) as the outcome variable* | | |  |  |  |  |
| Benefit-expansion reforms | 1.399^***^(0.451) | 1.785^***^(0.496) | 1.326^***^(0.471) | 1.692^***^(0.543) | 2.014(1.237) | 2.524(1.549) |
| Cost-containment reforms | -0.573(0.988) | 0.416(1.182) | -0.721(1.008) | 0.098(1.177) | 7.830(5.921) | 8.864(6.624) |
| Benefit-expansion*Cost-containment |  | -1.299^**^(0.519) |  | -1.101^**^(0.527) |  | -11.872(21.903) |
| Service delivery reforms | 0.075^**^(0.037) | 0.068^*^(0.037) | 0.079^**^(0.039) | 0.071^*^(0.039) | -0.055(0.087) | -0.055(0.087) |
| Pharmaceutical reforms | -0.252^***^(0.090) | -0.238^***^(0.091) | -0.274^***^(0.100) | -0.257^**^(0.101) | 0.073(0.177) | 0.073(0.176) |
| Days of inability to perform normal activities due to illness | 0.079^***^(0.008) | 0.079^***^(0.008) | 0.075^***^(0.009) | 0.075^***^(0.009) | 0.085^***^(0.016) | 0.086^***^(0.016) |
| Severity of illness | 0.318^***^(0.066) | 0.320^***^(0.066) | 0.304^***^(0.082) | 0.306^***^(0.082) | 0.356^***^(0.112) | 0.351^***^(0.113) |
| Chronic conditions | 0.265^*^(0.156) | 0.264^*^(0.155) | 0.099(0.163) | 0.097(0.163) | 1.253^***^(0.465) | 1.260^***^(0.465) |
| Health services utilization | 1.253^***^(0.097) | 1.259^***^(0.097) | 1.345^***^(0.122) | 1.351^***^(0.122) | 1.181^***^(0.163) | 1.186^***^(0.164) |
| Age | 0.013^***^(0.003) | 0.014^***^(0.003) | 0.011^**^(0.004) | 0.011^***^(0.004) | 0.009(0.007) | 0.009(0.007) |
| Schooling years | 0.009(0.012) | 0.009(0.012) | -0.002(0.015) | -0.002(0.015) | 0.038(0.026) | 0.039(0.026) |
| Household size | -0.050(0.039) | -0.049(0.039) | -0.026(0.051) | -0.025(0.051) | -0.066(0.060) | -0.070(0.060) |
| Log (per capita household income) | 0.066^*^(0.040) | 0.067^*^(0.040) | 0.008(0.064) | 0.008(0.064) | 0.090^*^(0.047) | 0.088^*^(0.047) |
| Constant | 1.776^***^(0.501) | 1.754^***^(0.501) | 3.262^***^(0.794) | 3.156^***^(0.751) | 1.548^**^(0.763) | 1.563^**^(0.766) |
| Year fixed effects | Y | Y | Y | Y | Y | Y |
| R2 | 0.303 | 0.304 | 0.293 | 0.294 | 0.271 | 0.271 |
| Observations | 1,429 | 1,429 | 959 | 959 | 470 | 470 |

Notes: ^*^*p* < 0.10, ^**^*p* < 0.05, ^***^*p* < 0.01. Robust standard errors in parentheses. In modelling medical spending that have limited values, two potential specifications can be used for analyses: two-part model (2PM) and Heckman selection model (HSM). 2PM assumes that seeking treatment and the choice of how much to spend are independent and discrete decisions for individuals, while HSM assumes that both decisions are affected by distinct but correlated observable and unobservable factors. To decide which model was more appropriate for modelling OOP spending, we first performed the HSM and generated the inverse Mills’ ratio (IMR) by conducting a probit regression of the probability of having positive OOP spending. We then tested for collinearity between other regressors and IMR by regressing OOP spending on both of them. When regressing OOP spending on IMR, benefit-expansion and cost-containment reforms and other covariates, we found that the coefficient of IMR was not significant (p = 0.187), suggesting the occurrence of OOP spending and the choice of how much to spend out-of-pocket may be independent. Moreover, IMR and other regressors were highly correlated (variance inflation factor = 14.580; R^2^ = 0.984 in the regressions of IMR on other regressors), suggesting that the HSM may be subject to unknown bias. Therefore, we used the 2PM instead of the HSM for robustness tests. Part 1 demonstrates probit regression results for whether an individual have positive OOP spending (OOP spending = 0 or not); Part 2 shows linear regression results for the determinants of how much OOP spending was, conditional on an individual having positive OOP spending (OOP spending > 0).

**Table C.** Robustness tests of the effects of UEBMI enrollment on OOP spending using the two-part models

|  | UEBMI enrollment | UEBMI enrollment* Policy reforms | |
| --- | --- | --- | --- |
|  | Model 7 | Model 8 |  |
| *Part 1: occurrence of OOP spending as the outcome variable* | | |  |
| UEBMI enrollment | -0.049(0.067) | 0.011(0.074) |  |
| Benefit-expansion reforms |  | 0.928^**^(0.424) |  |
| Cost-containment reforms |  | 7.112^**^(3.336) |  |
| UEBMI enrollment*Benefit-expansion |  | -0.685(0.464) |  |
| UEBMI enrollment*Cost-containment |  | -6.409^*^(3.369) |  |
| Benefit-expansion*Cost-containment |  | -20.489(12.762) |  |
| UEBMI enrollment*Benefit-expansion*Cost-containment |  | 20.282(12.763) |  |
| Service delivery reforms | 0.019(0.018) | 0.017(0.018) |  |
| Pharmaceutical reforms | -0.067(0.047) | -0.075(0.047) |  |
| Days of inability to perform normal activities due to illness | -0.033^***^(0.008) | -0.033^***^(0.008) |  |
| Severity of illness | 1.099^***^(0.034) | 1.093^***^(0.034) |  |
| Chronic conditions | -0.078(0.107) | -0.076(0.107) |  |
| Health services utilization | 1.389^***^(0.071) | 1.405^***^(0.072) |  |
| Age | -0.004^**^(0.002) | -0.004^**^(0.002) |  |
| Schooling years | -0.002(0.007) | -0.003(0.007) |  |
| Household size | -0.063^***^(0.018) | -0.064^***^(0.018) |  |
| Log (per capita household income) | 0.008(0.019) | 0.009(0.019) |  |
| Constant | -2.065^***^(0.222) | -2.067^***^(0.223) |  |
| Year fixed effects | Y | Y |  |
| Pseudo R^2^ | 0.573 | 0.575 |  |
| Observations | 13,033 | 13,033 |  |
| *Part 2: conditional OOP spending (in log) as the outcome variable* | | |  |
| UEBMI enrollment | 0.382^***^(0.129) | 0.470^***^(0.144) |  |
| Benefit-expansion reforms |  | 1.512(1.383) |  |
| Cost-containment reforms |  | 7.909(6.620) |  |
| UEBMI enrollment*Benefit-expansion |  | 0.298(1.434) |  |
| UEBMI enrollment*Cost-containment |  | -7.894(6.693) |  |
| Benefit-expansion*Cost-containment |  | -2.357(24.133) |  |
| UEBMI enrollment*Benefit-expansion*Cost-containment |  | 1.186(24.130) |  |
| UEBMI enrollment + UEBMI enrollment*Benefit-expansion |  | 0.768(1.396) |  |
| UEBMI enrollment + UEBMI enrollment*Cost-containment |  | -7.423(6.672) |  |
| UEBMI enrollment + UEBMI enrollment*Benefit-expansion + UEBMI enrollment*Cost-containment + UEBMI enrollment*Benefit-expansion*Cost-containment |  | -5.940(20.440) |  |
| Service delivery reforms | 0.072^**^(0.036) | 0.066^*^(0.037) |  |
| Pharmaceutical reforms | -0.247^***^(0.089) | -0.234^**^(0.093) |  |
| Days of inability to perform normal activities due to illness | 0.078^***^(0.008) | 0.077^***^(0.008) |  |
| Severity of illness | 0.318^***^(0.066) | 0.323^***^(0.066) |  |
| Chronic conditions | 0.231(0.157) | 0.249(0.155) |  |
| Health services utilization | 1.245^***^(0.097) | 1.283^***^(0.098) |  |
| Age | 0.010^***^(0.004) | 0.010^***^(0.004) |  |
| Schooling years | 0.004(0.013) | 0.002(0.013) |  |
| Household size | -0.034(0.039) | -0.037(0.039) |  |
| Log (per capita household income) | 0.052(0.040) | 0.053(0.039) |  |
| Constant | 2.025^***^(0.504) | 1.998^***^(0.504) |  |
| Year fixed effects | Y | Y |  |
| R^2^ | 0.299 | 0.310 |  |
| Observations | 1,429 | 1,429 |  |

Notes: ^*^*p* < 0.10, ^**^*p* < 0.05, ^***^*p* < 0.01. Robust standard errors in parentheses.
